# Evaluation of the effect of a comprehensive multidisciplinary care pathway for hip fractures: design of a controlled study

**DOI:** 10.1186/1471-2474-14-291

**Published:** 2013-10-12

**Authors:** Elvira R Flikweert, Gerbrand J Izaks, Inge HF Reininga, Klaus W Wendt, Martin Stevens

**Affiliations:** 1 Department of Surgery-Traumatology, University of Groningen, University Medical Center Groningen, P.O. Box 30.001, 9700 RB, Groningen, The Netherlands; 2 University Center for Geriatric Medicine, University of Groningen, University Medical Center Groningen, Groningen, The Netherlands; 3 Department of Orthopedics, University of Groningen, University Medical Center Groningen, Groningen, The Netherlands

**Keywords:** Hip fracture, Care pathway, Propensity score, Elderly, Physical functioning

## Abstract

**Background:**

Hip fractures constitute an economic burden on healthcare resources. Most persons with a hip fracture undergo surgery. As morbidity and mortality rates are high, perioperative care leaves room for improvement. Improvement can be achieved if it is organized in comprehensive care pathways, but the effectiveness of these pathways is not yet clear. Hence the objective of this study is to compare the clinical effectiveness of a comprehensive care pathway with care as usual on self-reported limitations in Activities of Daily Living.

**Methods/Design:**

A controlled trial will be conducted in which the comprehensive care pathway of University Medical Center Groningen will be compared with care as usual in two other, nonacademic, hospitals. In this trial, propensity scores will be used to adjust for differences at baseline between the intervention and control group. Propensity scores can be used in intervention studies where a classical randomized controlled trial is not feasible. Patients aged 60 years and older will be included. The hypothesis is that 15% more patients at University Medical Center Groningen compared with patients in the care-as-usual condition will have recovered at least as well at 6 months follow-up to pre-fracture levels for Activities of Daily Living.

**Discussion:**

This study will yield new knowledge with respect to the clinical effectiveness of a comprehensive care pathway for the treatment of hip fractures. This is relevant because of the growing incidence of hip fractures and the consequent massive burden on the healthcare system. Additionally, this study will contribute to the growing knowledge of the application of propensity scores, a relatively novel statistical technique to simulate a randomized controlled trial in studies where it is not possible or difficult to execute this kind of design.

**Trial registration:**

Nederlands Trial Register NTR3171

## Background

Hip fractures constitute an economic burden on healthcare resources that will continue to rise. Approximately 17,000 older persons sustain a hip fracture in the Netherlands every year [1]. Costs of treatment, rehabilitation and home care are considerable (nearly EUR 400 million per year). Regardless of the costs, hip fractures have a negative effect on activities of daily living and quality of life. One year after the fracture, less than 50% of patients have fully recovered [2]. Most persons with a fracture undergo surgery. However, perioperative care in hip fracture patients leaves much room for improvement, as convalescence after surgery is often hindered by complications. As a consequence, hip fracture is associated with high mortality and considerable loss of function [3]. Complications may occur in many organ systems and require the involvement of various medical specialties. In theory, the involvement of many specialties should guarantee the best possible treatment, yet in practice the care tends to be rather fragmented. Therefore, it is generally felt that perioperative care can be improved if care is organized in comprehensive care pathways. Aim of care pathways is to optimize care during the entire process of surgery and rehabilitation. The effectiveness of care pathways for persons with a hip fracture was investigated in studies that show a tendency for the pathways being more effective than usual care [4-6]. However, the extensiveness of these care pathways differs and most care pathways deal with only one or a few aspects of the care (e.g. the postoperative rehabilitation in hospital care).

To overcome this shortcoming a comprehensive care pathway for the treatment of patients with a hip fracture was developed at University Medical Center Groningen (UMCG). In this pathway the care is described from arrival at the emergency room until discharge from the nursing home. The objective of this study is to determine the clinical effectiveness of this pathway. To that end, a controlled trial will be conducted in which the new comprehensive care pathway of UMCG will be compared with care as usual at Martini Hospital Groningen (MHG) and Ommelander Hospital in Winschoten (OHW). It is hypothesized that 15% more patients treated in the new comprehensive care pathway compared with patients in the care-as-usual condition will have recovered at least as well at 6 months follow-up to pre-fracture levels in terms of Activities of Daily Living (ADL). As a randomized controlled trial (RCT) is not feasible, propensity scores will be used to adjust for differences at baseline between the intervention and control group.

## Methods/Design

### Study design

A controlled intervention will be executed to compare the clinical effectiveness of the new comprehensive care pathway for the treatment of hip fractures with care as usual. Initially a RCT was planned for this purpose. A RCT within one hospital was not justified, because patients are clustered in one ward with a dedicated team. To treat patients with the same diagnosis in two different ways, it was decided to compare patients to be treated with the care pathway at UMCG with care as usual in other hospitals in the Groningen area. To assess if such a RCT was feasible a pilot study was done to test the randomization process. This showed that randomization on the spot performed by ambulance personnel was not feasible. Of the 154 elderly patients with a hip fracture eligible for randomization, only 14 (9%) gave informed consent. It was therefore decided to perform a controlled trial with propensity score adjustment. Propensity scores can be used to adjust for differences at baseline between the intervention and control group in situations in which a classical RCT is not feasible.

The study has been approved by the Medical Ethical Committee of University Medical Center Groningen and is registered in the Dutch trial register (NTR3171).

### Setting

The study will be executed in three hospitals in the northeastern part of the Netherlands. UMCG is a tertiary university hospital, located in the city center of Groningen. Martini Hospital Groningen (MHG) is a large teaching hospital in the southern part of the city and Ommelander Hospital Winschoten (OHW) is a smaller, rurally-located hospital situated in the town of Winschoten, 40 km east of the city of Groningen. Although these three hospitals differ in certain aspects such as size, medical specialty services and educational assignments, they all offer the same standard care for patients with a hip fracture. In the Netherlands, standard care for patients with a hip fracture is based on a national professional guideline [7]. Therefore, it can be assumed that the usual care in the three hospitals prior to the intervention study is comparable. Patients are taken to one of the hospitals on basis of proximity and availability, not based on the characteristics of the hospital. Especially with respect to the UMCG, it must be mentioned that this hospital does not only provide academic or tertiary care but due to its position as one of the few hospitals in the Northern Netherlands, also provides general (secondary) medical and surgical care. Patients admitted to UMCG for treatment will be treated in the new comprehensive care pathway. Patients transported to Martini Hospital Groningen and Ommelander Hospital Winschoten will receive care as usual.

### Participants

Patients aged 60 years and older with a hip fracture defined as a femoral neck fracture (dislocated or not dislocated) and pertrochanteric and intertrochanteric fractures (AO Comprehensive Classification 31.A.1; 31.A.2; 31.A.3) will be included. Exclusion criteria are patients with multi-trauma injuries (thoracic and/or abdominal) and patients who are not able to fill in questionnaires, who are not able to understand the Dutch language, or who are not able to give informed consent. Patients with mild dementia, who do not fully understand the study, but are able to fill in the questionnaires with the help of close relatives can be included when these close relatives give their informed consent and the patient agrees.

### Intervention

In the new comprehensive care pathway of UMCG the treatment and the role of every participant is described from arrival at the emergency room until discharge from the nursing home. This new treatment protocol comprises multidisciplinary cooperation between trauma surgeon, orthopedic surgeon, anesthesiologist and geriatrician, a preoperative workup protocol, a fixed moment of surgery and a postoperative protocol in close collaboration with the physical therapist and nursing home physician. The comprehensive care pathway does not end with discharge from the hospital. The core elements of the pathway are:

– A shared preoperative planning protocol for both the departments of orthopedics and traumatology.

– A perioperative anesthesiological risk assessment in the emergency room already, instead of later on the ward.

– Enrolment of the patients for surgery at a fixed time in the emergency operating rooms (OR). Each morning at 8:00 AM an emergency room with plenum is available for such a patient if necessary. An experienced surgeon and OR team are also available.

– Because of the fixed time of surgery, 8:00 AM on the day after admission, there is allowance for patients to eat until midnight — eight hours before surgery — causing minimal discomfort.

– A uniform treatment protocol for both surgery teams of the departments of orthopedics and traumatology.

– Clustering of all hip fracture patients on one nursing ward in order to use the experience and knowledge of the nursing staff and consequently improve the quality of care for the patient, with extra attention for care of elderly patients (early start of rehabilitation, adequate nutrition, prevention of pressure ulcers and delirium).

– Daily visits by the geriatric team during the stay at the nursing ward.

– Early initiation of standard discharge procedures. Upon arrival at the hospital the patient is already registered for transfer to a nursing home.

– Planned capacity at the nursing homes for an efficient transfer of patients. In addition, the nursing home physician is able to view the data of patients with hip fracture in the data management systems of UMCG. Since this doctor handles the admissions, he/she can view the progress of the patients and anticipate their arrival.

– Patients are seen at a special outpatient clinic for hip fracture patients. The last visit is planned 6 months after the operation. Special attention is paid to functioning in daily living activities.

### Care as usual

At Martini Hospital Groningen and Ommelander Hospital Winschoten, patients will receive care as usual. This comprises the following:

– The diagnosis of hip fracture is established by assessment and X-ray at the emergency room.

– Depending on the general surgeon or orthopedic surgeon on call (fixed rotation schedule), a decision about the operative treatment is made. The choice for a specific surgical procedure depends on the surgeon on call and the local trauma protocol.

– The patient will be admitted to one of the surgical or orthopedic wards.

– Preoperatively an anesthesiologist will examine the patient and assess available medical information.

– The patient will be treated according to local hospital protocols and techniques.

– From the day of admission specific risk factors (malnutrition, decubitus risk, delirium) are categorized in a standardized way using a scoring list and specialized staff.

– A geriatrician is not involved by default.

– Postoperatively the patient will be mobilized, and physical therapy is arranged for.

– There are some agreements with nursing homes for transfer to a rehabilitation facility or retirement center.

### Measurements

Measurements will be taken preoperatively, perioperatively, and at 6 weeks and 3 and 6 months after surgery. Preoperative demographic data, preoperative diagnosis, height, weight, Body Mass Index and ASA classification will be recorded. Hip fractures will be classified as femoral neck fractures and pertrochanteric or intertrochanteric fractures. Average surgical time, intraoperative blood loss, in-hospital transfusion rate and complications will be recorded perioperatively. Mortality will also be recorded.

Self-reported limitations in ADL will be measured with the Katz index and Lawton Instrumental Activities of Daily Living (IADL) scoring list [8,9]. The Katz index is based on an evaluation of the functional dependence or independence of patients in bathing, dressing, going to the toilet, transferring, continence and feeding. ADL index A indicates independence in all six functions, index B independence in all but one of the six functions. Indexes C–G indicate dependence in bathing and at least one additional function. The IADL scoring list is an evaluation of the abilities of patients in eight activities: using the telephone and transportation, shopping, preparing food, housekeeping and laundry, responsibility for their own medications and handling finances. On the basis of the score patients are divided into different categories that state something about their degree of independence.

Health-related quality of life will be measured using the EQ-5D (EQ-5D™ Dutch© 1987 Euroquol Group). The EQ-5D has five dimensions: mobility, self-care, usual activities, pain/discomfort, and anxiety/depression. Each dimension is divided into three degrees of severity: no problem, some problems, and major problems. Actual quality of life must also be signed on the EQ VAS scale. The Katz index and IADL scoring list are measured at the hospital (to determine the situation before the fracture) and at 6 weeks and 6 months after the operation. The EQ-5D will be assessed on the same occasions and at 3 months after the operation.

At the outpatient clinic, at 6 weeks and 6 months after the operation, fracture consolidation, complications and Harris Hip Score will be assessed [10]. The Harris Hip Score (HHS) is a validated functional hip score that measures four items: pain, function, range of motion and deformity, in that order of importance. A score between 90 and 100 points means an excellent outcome, a score lower than 70 means a poor outcome. Six months after the operation the living and independence situation (percentage of people living in their own home again) of the patient is evaluated.

### Sample size

The power calculation is based on the hypothesis that 15% more patients treated with the new comprehensive care pathway compared with patients in the care-as-usual condition will have recovered at least as well at 6 months follow-up to pre-fracture levels in terms of ADL as measured with the Katz index. In order to detect this 15% difference with 80% power at a significance level of 0.05 (one-sided), 130 patients are needed in the new comprehensive care pathway condition and 130 in the usual care condition. Since a comparison will be made between UMCG and MHG as well as between UMCG and OHW, 130 patients will be included in MHG and 130 in OHW. Taking into account an expected drop out-rate of 15% a total of 450 patients have to be included.

### Statistical analyses

Statistical analysis will be performed with IBM SPSS Statistics 19.0 (IBM, Amonk, NY). As conventional randomization is not feasible in this patient group, propensity score adjustment will be applied. Propensity scores, or the likelihood to be in the treatment condition or control condition, will be estimated with the following (pre-fracture) variables: age, gender, marital status, living situation, functional status, co morbidity, cognitive function, number of medications, need for home care, need for day care. Propensity scores will be used as an independent variable in the multivariate analysis.

Descriptive statistics will be used to describe the main characteristics of the population. For comparison between UMCG and MHG and OHW a multivariate analysis of variance (MANOVA) will be applied. The Katz index, Lawton Instrumental Activities of Daily Living (IADL) scoring list, the EQ-5D and HHS will be included as dependent variables and hospital and the propensity score as independent variables. A p-value lower than 0.05 will be considered as statistically significant.

## Discussion

Based on available reviews in the literature, it can be concluded that there is no level-1 evidence for the effectiveness of comprehensive care pathways for elderly patients with a hip fracture [11,12]. Also this study will not reveal level-1 evidence, however it will reveal new knowledge on the clinical effectiveness of a comprehensive care pathway for the treatment of hip fractures. This is relevant because of the growing incidence of hip fractures and the ensuing massive burden on the healthcare system. So far, there is no definitive answer as to whether a care pathway is better than usual care [11]. Non-randomized studies into the effectiveness of pathways have reported promising results in decreasing waiting time, length of hospital stay and complication rate [13-18]. However, a major drawback is that they are only dealing with one or a few aspects of care. A strong point of the current research is the fact that the clinical effectiveness of a comprehensive care pathway is subject of study. The rehabilitation of a hip fracture patient does not stop after discharge from the surgical ward, but continues for at least six months. It is thus important to include all the aspects of care and all involved disciplines in the care pathway, from the nurse in the emergency room to the doctor at the nursing home.

In addition, this study will also reveal new knowledge on the application of propensity scores, a novel statistical tool to simulate RCTs in situations in which a classical RCT is not feasible. As already mentioned, there is no definitive answer yet as to whether a care pathway is more effective than usual care. Especially with respect to comprehensive care pathways, this evidence is lacking. An important issue in this respect is the fact that RCTs are difficult to perform in hip fracture patients, who are characterized by advanced age and frailty. In general, a RCT is considered the highest standard for determining the effectiveness of a new intervention. However, it is not always feasible to use random assignment. For such situations, propensity scores are considered an useful alternative [19,20]. With propensity scores, adjustments can be made for observed variables to minimize or remove the bias, which normally disturbs the results in observational studies. When the observed covariates are on average balanced between exposure groups, the advantages of a randomization process are approached, except that it is not possible to adjust for unobserved covariates [21]. However, studies comparing RCTs and observational studies using propensity scores have shown that the method is reliable. This is the reason why propensity scores has been used more often in recent times and why it will be used in this study [22].

## Abbreviations

HHS: Harris hip score; (I)ADL: (Instrumental) activities of daily living; MHG: Martini Hospital Groningen; OHW: Ommelander Ziekenhuis Winschoten; OR: Operating room; RCT: Randomized controlled trial; UMCG: University Medical Center Groningen.

## Competing interests

Financial competing interest: the study is supported by a grant from Biomet and Traumacentrum Noord Nederland.

## Authors’ contributions

EF wrote the manuscript and designed the study. GI designed the study and reviewed the manuscript. IR contributed on statistics and epidemiology. KW designed the study, reviewed the manuscript, is head of the department and arranged the finances. MS designed the study and was co-writer of the manuscript. All authors read and approved the final manuscript.

## Pre-publication history

The pre-publication history for this paper can be accessed here:

http://www.biomedcentral.com/1471-2474/14/291/prepub
